# Minimal Invasive Surgical Treatment in Primary Sacrococcygeal Pilonidal Sinus Disease: Phenolization of the Sinus Tract Versus Sinus Laser Assisted Closure; Long‐Term Follow‐Up of a Comparative Cohort Study

**DOI:** 10.1002/wjs.70028

**Published:** 2025-08-06

**Authors:** Jochem de Kort, Laurens V. van Kempen, Ruben Schouten, Menno R. Vriens, Niels Smakman, Edgar J. B. Furnee

**Affiliations:** ^1^ Department of Surgery Diakonessenhuis Utrecht the Netherlands; ^2^ Department of Surgery Flevoziekenhuis Almere the Netherlands; ^3^ Department of Surgery University Medical Center Utrecht Utrecht the Netherlands; ^4^ Department of Abdominal Surgery University Medical Center Groningen Groningen the Netherlands

**Keywords:** colorectal disease, laser treatment, phenolization, sacrococcygeal pilonidal sinus disease

## Abstract

**Background:**

No consensus has been reached on the preferred minimally invasive technique for treating primary, chronic sacrococcygeal pilonidal sinus disease. This study will compare two extensively used minimally invasive techniques: phenolization of the sinus tract and sinus laser‐assisted closure.

**Methods:**

A comparative cohort study was conducted in two hospitals. Patients were matched on sex, age, number of sinus pits at surgery, and duration of follow‐up. Patient records were examined for additional surgeries, and quality of life and symptoms were assessed with a questionnaire.

**Results:**

After a mean follow‐up of 47 months, seven patients (19.4%) in the phenolization group (*n* = 36) required additional surgery and 11 patients (30.6%) in the SiLaC group (*n* = 36) underwent additional surgery. Pain as a symptom at the natal cleft was significantly lower in the phenolization group, though not clinically relevant. The question regarding general health compared to preoperatively was reported to be significantly better in the phenolization group. However, other quality of life outcomes, such as the VAS score and the SF‐36, did not differ significantly between phenolization and SiLaC.

**Conclusion:**

Both phenolization and sinus laser‐assisted closure appear to be feasible for patients with primary SPSD, with no clear superiority of either technique.

## Graphical abstract

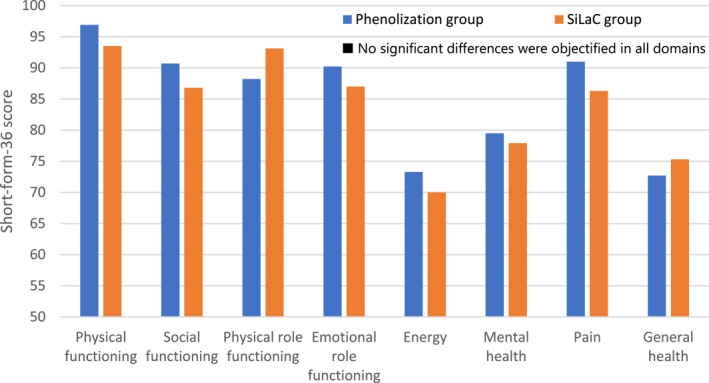

## 1 Introduction

Pilonidal sinus disease is an inflammatory, benign condition that primarly affects the sacrococcygeal region. Historically, the treatment of sacrococcygeal pilonidal sinus disease (SPSD) consisted primarily of excisional techniques, such as wide excision with either direct (off‐) midline wound closure or secondary wound healing, the Karydakis procedure [1], the Bascom cleft lift procedure [2], or various flap reconstructions [3]. However, with the introduction of the pit‐picking technique [4], several other minimally invasive techniques have since been developed. Three minimally invasive techniques are currently available, including endoscopic treatment (video‐assisted ablation of pilonidal sinus, VAAPS, or endoscopic pilonidal sinus treatment, EPSiT) [5, 6], phenolization of the sinus tract [7], and sinus laser‐assisted closure (SiLaC) [8]. These techniques share the general principles, including the removal of hair and debris, as well as de‐epithelialization of the sinus tract. Nowadays, for simple, chronic SPSD, one of these minimally invasive techniques should be considered as the primary treatment before opting for a more extensive surgical excision approach, due to the proven advantages of these minimally invasive techniques, such as less pain, faster wound healing, and quicker return to normal activities [9]. However, there is still no consensus on whether one minimally invasive technique should be preferred.

The phenolization technique and SiLaC are the most widely used minimally invasive techniques. Although considerable research has been conducted on each technique independently, direct comparative studies remain scarce, with only two retrospective comparative studies published to date. One study reported a shorter operative time in the phenolization group (PG), whereas both studies observed less pain in the SiLaC group (SG). No significant differences in recurrence rates were observed, which may be attributable to the short follow‐up periods of only six and 12 months, respectively [10, 11]. Consequently, long‐term data comparing the phenolization technique and SiLaC are essentially lacking in the literature.

The aim of this comparative cohort study was to compare the long‐term outcome of the phenolization technique and SiLaC, focusing on additional interventions, pilonidal‐related symptoms, and quality of life.

## 2 Methods

### 2.1 Patient Population

The data for this comparative cohort study were obtained from two Dutch hospitals. Data for patients who underwent the phenolization procedure were extracted from a preexisting database at the Diakonessenhuis in Utrecht, the Netherlands [12]. The patients who were randomized to the phenolization technique in the previously published randomized trial comparing the phenolization technique with local excision followed by primary wound closure, and in whom long‐term follow‐up was available, were included in the phenolization cohort for the current study. Those patients were compared to a cohort of patients treated with SiLaC, selected from a retrospectively collected database at the Flevoziekenhuis in Almere, the Netherlands. Matching of the PG and the SG was based on sex, age, number of sinus pits at surgery, and duration of follow‐up. The inclusion criteria for both study groups encompassed patients with primary SPSD who were older than 18 years. Patients were excluded if they had recurrent SPSD, a pilonidal abscess, or extensive subcutaneous tract formation, as these presentations are less suitable for minimally invasive techniques. Informed consent was obtained from all participants prior to enrollment. Ethical approval was granted by the medical ethics committee in Utrecht, the Netherlands.

### 2.2 Surgical Techniques

In the phenolization technique, patients were positioned prone. The midline sinus pits and, if present, off‐midline drainage orifices, were excised to a limited extent, and curettage was performed on the sinus tracts to remove hair, debris, and granulation tissue. After protecting the surrounding tissue with vaseline, liquid phenol (85%) was applied to the sinus tracts for 1 minute, twice. The phenol was neutralized with ethanol, and the wound was left open [13]. In patients who received SiLaC, patients were positioned sideways. The midline pits were probed to determine the extent of the underlying tract. Curettage was then performed to remove hairs, debris, and granulation tissue. A laser probe was fully inserted, activated, and slowly retracted (approximately 1 mm per second), causing the tract to shrink and close. The amount of joules administered was determined by the length of the sinus tract present. If the tract did not fully close, the probe was reinserted for a second application.

### 2.3 Data Collection

Demographic data, including sex, age, BMI, occupation, number of sinus pits objectified during surgery, and smoking status were prospectively collected for the PG as part of the randomized controlled trial [13] and retrospectively from the patients' records for the SG.

For long‐term follow‐up, a questionnaire, similar to the one used in the PG as part of the long‐term follow‐up of the randomized controlled trial [12], was also sent to the patients included in the SG. In this questionnaire, several well‐known pilonidal‐related symptoms were assessed, including fluid discharge, pain, itching, irritation, and burning sensation. These symptoms were evaluated using a six‐point Likert scale (0 indicating no complaints, five indicating daily complaints). Patients were also asked to rate their general health status compared to their preoperative condition on a three‐point scale: better, the same or worse. In addition, the current disease status compared to the preoperative disease status was assessed on a four‐point scale: completely healed, better but not completely healed, the same, or worse. Patients were also asked how beneficial they found the treatment and whether they would choose the same treatment again if given the option. Finally, the burden of the treatment was assessed with a VAS score, ranging from 0 to 100, with lower scores indicating less burden of treatment.

Quality of life was assessed using the Short Form(SF)‐36 [14], a specially designed questionnaire to evaluate health‐related quality of life, and also by a VAS score, with 0 meaning low quality of life and 100 meaning the best quality of life.

Finally, the patients were asked whether they had undergone surgery for recurrent SPSD after the index operation at another hospital or whether they had symptoms of a persistent or recurrent SPSD. In addition to this question, all patient records were reviewed to detect any objective recurrence, complications, and/or the need for an additional surgical intervention following the index operation for recurrent SPSD.

### 2.4 Statistical Analysis

Analysis was carried out with SPSS statistics version 29.0.2 for Windows. A Kolmogorov–Smirnov test was conducted to assess whether baseline characteristics were normally distributed. If data were normally distributed, values were expressed as mean with standard deviation. If data were not normally distributed, values were expressed as median with the interquartile range. For the analysis of statistical significance of categorical variables, a Fisher's exact test or the chi‐squared test was performed. An independent samples *t*‐test or a Mann–Whitney *U* test was conducted to compare continuous data between groups, depending on the data distribution. To compare paired data, a Wilcoxon rank‐sum test was conducted. Significance was set at *p* < 0.05.

## 3 Results

The phenolization cohort included the 36 patients for whom long‐term follow‐up data within the previously mentioned randomized controlled trial were available [12]. This group was matched with 36 patients selected from the retrospectively collected database of patients who underwent SiLaC, based on the aforementioned parameters. Baseline characteristics and follow‐up times for both groups are presented in Table 1.

**TABLE 1:** Baseline characteristics.

	Phenolization (*n* = 36)	SiLaC (*n* = 36)	*p* value
Male sex (%)	31 (86.1)	31 (86.1)	1.000
Age (years)	31.0 (IQR 28 – 37)	31.5 (IQR 25.5 – 44.5)	0.813
Body mass index (kg/m^2^)	24.9 (IQR 22.6 – 28.0)	25.0 (IQR 23.2 – 28.8)	0.783
Sinus pits at surgery (*n*)	2 (IQR 1 – 3.25)	2 (IQR 1 – 4)	0.875
Smoking (%)	6 (16.7)	5 (13.9)	0.743
Family history of SPSD (%)	6 (16.7)	7 (19.4)	0.759
Working in sitting position (%)	29 (80.6)	27 (75.0)	0.571
Duration of preoperative symptoms (months)	6.5 (IQR 3.0 – 14.0)	8.5 (IQR 2.3 – 33.0)	0.520
Duration of follow‐up (months)	48.2 (± 12.7)	45.3 (± 10.4)	0.358

*Note:* Values are reported as percentage, as mean (± standard deviation), or as median (interquartile range).

Abbreviation: SPSD, sacrococcygeal pilonidal sinus disease.

### 3.1 Subjective Outcomes

Data on subjective long‐term outcomes for both groups are reported in Table 2. At long‐term follow‐up, all SPSD‐related symptoms at the natal cleft showed no significant differences between the two groups, except for pain.

**TABLE 2:** Subjective long‐term outcome.

	Phenolization (*n* = 36)	SiLaC (*n* = 36)	*p* value
**Symptoms at natal cleft** ^a^			
Fluid discharge	0.0 [0.0 – 0.0]	0.0 [0.0 – 0.0]	**0.356**
Itch	0.0 [0.0 – 0.0]	0.0 [0.0 – 1.0]	**0.157**
Pain	0.0 [0.0 – 0.0]	0.0 [0.0 – 1.0]	**0.032**
Irritation	0.0 [0.0 – 0.0]	0.0 [0.0 – 0.0]	**0.325**
Burning sensation	0.0 [0.0 – 0.0]	0.0 [0.0 – 0.0]	**0.307**
General health compared to preoperatively			**0.048**
Better (%)	22 (61.1)	15 (41.7)	
The same (%)	11 (30.6)	18 (50.0)	
Worse (%)	1 (2.8)	3 (8.3)	
Missing (%)	2 (5.6)	0 (0.0)	
Status of disease compared to preoperatively			**0.239**
Completely healed (%)	25 (69.4)	22 (61.1)	
Better, but not completely healed (%)	8 (22.2)	11 (30.6)	
The same (%)	1 (2.8)	3 (8.3)	
Worse (%)	0 (0.0)	0 (0.0)	
Missing (%)	2 (5.6)	0 (0.0)	
Treatment considered beneficial			**0.645**
Very much (%)	26 (72.2)	26 (72.2)	
Quite a bit (%)	5 (13.9)	5 (13.9)	
A little bit (%)	2 (5.6)	4 (11.1)	
Not at all (%)	1 (2.8)	1 (2.8)	
Missing (%)	2 (5.6)	0 (0.0)	
Choosing the same treatment again			**0.602**
Yes (%)	30 (83.3)	30 (83.3)	
Indifferent (%)	3 (8.3)	3 (8.3)	
No (%)	1 (2.8)	3 (8.3)	
Missing (%)	2 (5.6)	0 (0.0)	
Burden of treatment (VAS‐score)	20 [10 – 46.5]	50 [19.3 – 61.5]	**0.055**

*Note:* Values are reported as median and interquartile range (IQR) unless otherwise stated. The bold *p*‐values indicate the level of significance between the two groups for each subjective outcome parameter.

Abbreviation: VAS, visual analog scale.

^a^ Items scored on a six‐point scale (0 meaning no complaints, 5 meaning daily complaints).

The question regarding general health compared to the preoperative period was scored significantly better in patients who were treated with phenolization (Table 2). However, the status of the disease compared to preoperatively, the effectiveness of the treatment, and the likelihood of choosing the same treatment again were all positively evaluated in both groups with no statistically significant differences between the two groups. Patients treated with phenolization reported a lower treatment burden than those treated with SiLaC. However, this finding did not reach statistical significance (Table 2).

**FIGURE 1:**
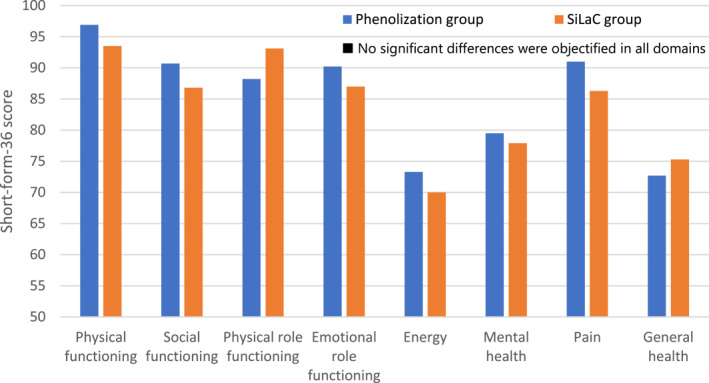
Short Form‐36 at long‐term follow‐up.

Quality of life at follow‐up, measured by the SF‐36, is depicted in Figure 1. Although patients in the PG scored higher in six out of eight SF‐36 domains, the difference was not statistically significant. The quality of life scores at follow‐up, measured on a visual analog scale, did not show a statistically significant difference between the PG and SG (*p* = 0.479).

### 3.2 Additional Surgery

**FIGURE 2:**
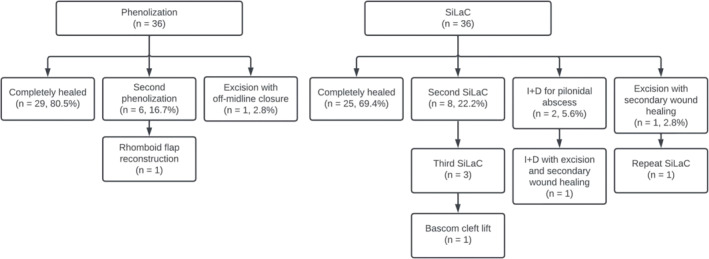
Flowchart of additional interventions per treatment modality.

In the PG, seven patients (19.4%) required an additional intervention following the index operation (Figure 2). Six patients (16.7%) underwent a second phenolization treatment due to incomplete wound closure. Mean time between the first and second phenolization procedure was 16.2 (± 7.4) months. After the second treatment, all wounds healed completely except in one patient. This patient underwent excision with rhomboid flap reconstruction at 27 months after the index operation because of recurrent SPSD, 24 months after the second phenolization procedure. Additionally, one other patient also required more extensive surgery: at 24 months post‐procedure, this patient underwent radical excision with off‐midline closure. All patients eventually achieved complete wound healing.

In the SG, 11 patients (30.6%) required additional surgery following the index operation (Figure 2). Eight patients (22.2%) underwent one additional SiLaC procedure due to incomplete wound closure and persistent symptoms. Mean time between the first and second SiLaC procedure was 10.0 (± 6.4) months. Three of these patients required another SiLaC procedure, performed 14 months (*n* = 2) and 21 months after their initial SiLaC procedure. One of these patients did not achieve complete wound healing and underwent a Bascom cleft lift. All other wounds healed after the second or third procedure. Additionally, two patients (5.6%) developed a pilonidal sinus abscess after the index operation; one patient was treated with incision and drainage once, 1 month after the index operation, and the other was treated with incision and drainage 31 months after the index operation and redeveloped an abscess 1 month later (i.e., 32 months after the index operation), which was managed with incision and drainage combined with limited excision of the sinus pits followed by secondary wound healing. One other patient required more extensive surgery and was treated with excision with secondary wound healing 11 months after the index operation, followed by another SiLaC 3 months later. The SPSD finally healed after these interventions.

There was no statistically significant difference in the number of patients requiring an additional intervention after the index operation, regardless of the type of additional intervention, between the SG and the PG (11 vs. 7, respectively, *p* = 0.276).

In the SG, four patients (11.1%) reported in the questionnaire that they had the impression of recurrent SPSD compared to none in the PG (*p* = 0.115). After contact, one patient was scheduled for an appointment and is awaiting treatment. The other patients could not be reached despite repeated attempts. Additionally, no patients in either cohort reported undergoing additional pilonidal sinus surgery at another hospital.

## 4 Discussion

This is, to our knowledge, the first study comparing the phenolization technique and SiLaC after long‐term follow‐up. Although there was no significant difference between most symptoms at the natal cleft at follow‐up between both groups, only pain at the natal cleft at follow‐up reached a significant difference between both treatments, favoring the phenolization technique. However, the absolute difference in pain scores was small and therefore does not seem to be clinically relevant. Additionally, the question regarding general health compared to preoperatively was reported to be significantly better in the PG. However, other outcomes of quality of life, such as quality of life measured by a VAS score and the SF‐36, did not significantly differ between phenolization and SiLaC. Other parameters analyzed, including the treatment burden and the need for additional surgery, also showed no significant differences between the two groups.

Only two other studies have been published comparing the phenolization technique with SiLaC treatment [10, 11]. Taskin et al. compared 40 patients who underwent phenolization with 40 patients who received SiLaC, whereas Emral et al. reported results from a cohort of 70 patients who underwent phenolization and 51 patients who received SiLaC. However, compared to the follow‐up time in the current study, the follow‐up durations in these studies were relatively short, with 6 and 12 months of follow‐up, respectively. Taskin et al. reported a significantly shorter operative time in the PG, and both studies found significantly lower pain scores in the SG. This contrasts with the findings in our study, where pain scores were significantly lower in the PG. This discrepancy may be attributable to differences in the timing of pain score evaluations. In our study, pain was evaluated at long‐term follow‐up, whereas in the other studies, pain was assessed within several hours to one day post‐intervention. Both studies reported no significant differences in recurrence rates at follow‐up, which aligns with our findings, where no statistically significant differences were found in the number of additional surgeries after the index operation between the two groups. Taskin et al. also evaluated patient satisfaction and reported no significant differences between the two treatment groups. Similarly, in our study, quality of life was assessed using the SF‐36, and no significant differences were observed between the two groups.

Cohort studies involving either the phenolization technique or SiLaC with follow‐up times comparable to those in the current study have been conducted previously. Karita et al. performed a retrospective cohort study involving 83 patients receiving SiLaC treatment for primary or recurrent SPSD, with a median follow‐up of 5.2 years [15]. This study represents the longest follow‐up data on SiLaC available in the literature to date. Among patients with primary disease (*n* = 70), they reported a reoperation rate of 20%. Patient satisfaction and quality of life were not assessed. In the current study, the reoperation rate in the SG was slightly higher at 30.6%. Although we do not have a clear explanation for this difference, it may be attributable to the small sample size.

Regarding treatment with phenolization, Calikoglu et al. conducted a randomized controlled trial comparing phenolization to excision [16]. Among patients treated with phenolization, a recurrence rate of 18.6% was observed after a follow‐up of 40 months. However, the study did not differentiate between primary and recurrent disease in its patient population. Their findings are comparable to our results, where we observed a reoperation rate of 19.4%. Quality of life was also assessed in their study, with mean scores for the physical and mental components of quality of life derived from the SF‐36 being 53.1 and 50.6, respectively. These scores were comparable to those reported in the current study for the PG: 54.2 for the physical component and 52.5 for the mental component [17].

Although pain at the natal cleft and general health compared to preoperatively were the only factors that reached a statistically significant difference between both groups, favoring the PG, other parameters analyzed in this study also showed a trend toward a favorable outcome in the PG. These parameters included the need for additional surgery, the total burden of the treatment, and six out of eight SF‐36 quality of life domains. The relatively limited sample size of both cohorts may explain why no statistical significance was found for these parameters. Moreover, although no cost‐effectiveness analysis was performed, the costs associated with SiLaC are likely higher than those of phenolization due to the use of resources required for surgery. For future research, a larger cohort study, preferably in a prospective setting and including a cost‐effectiveness analysis, should be considered to further evaluate potential differences in outcomes between both treatment options.

This study has some limitations. First, there was a difference in the study design between both treatment cohorts: The phenolization cohort had a prospective design, whereas the SiLaC cohort was retrospective. Since the SG and PG were treated in different hospitals, and so by different practitioners, some selection bias should be considered. For instance, no data on lateral drainage openings were available for analysis, which could possibly contribute to selection bias. However, the number of sinus pits at presentation was assessed in both groups, with no statistically significant difference observed between them. Although this finding reduces the risk of selection bias, some residual selection bias may still persist. Second, some confounding bias must be considered in this study. To minimize this, groups were matched on key confounders affecting surgical outcomes, such as age, sex, number of sinus pits, and follow‐up duration [4, 18]. Although this matching reduced the risk of confounding bias, some residual bias may still be present. Additionally, due to the retrospective design of the study, matching for another potential confounder, the amount of hair in the sacrococcygeal area, was not possible [19, 20]. Lastly, the small cohort size may have influenced the statistical significance of the results. Ideally, a large, randomized controlled trial comparing multiple minimally invasive techniques should be conducted to further validate our findings. However, this could be challenging, as many centers specialize in only one technique, which complicates randomization. As mentioned earlier, a large prospective comparative cohort study could determine whether the nonsignificant differences between the phenolization technique and SiLaC, as found in the current study, would reach statistical significance in a larger cohort.

## 5 Conclusion

This comparative cohort study of the phenolization technique and SiLaC in patients with primary SPSD, with a follow‐up duration of approximately 4 years, provides the longest follow‐up to date on these techniques in a comparative design. Symptom scores at the natal cleft were low in both groups, and, therefore, the difference in pain score between both groups does not seem to be clinically relevant. Although general health compared to preoperatively was significantly better in the PG, the other outcomes on quality of life, including the SF‐36 and VAS‐score, did not show statistically significant differences. The need for additional surgery and the total treatment burden showed favorable but nonsignificant trends toward the phenolization technique. Based on these findings, both techniques appear to be feasible for patients with primary SPSD, with no clear superiority of one technique over the other. However, especially due to the more favorable, though only partially statistically significant findings for the phenolization technique, further validation of these findings in a large prospective comparative cohort study is required.

## Author Contributions

**Jochem de Kort:** investigation, conceptualization, data curation, formal analysis, methodology, writing original draft. **Laurens V. van Kempen:** investigation, data curation, writing – review and editing. **Ruben Schouten:** investigation, data curation, writing – review and editing. **Menno R. Vriens:** methodology, supervision, interpretation, writing – review and editing, project administration. **Niels Smakman:** methodology, supervision, interpretation, writing review and editing, project administration. **Edgar J. B. Furnee:** methodology, supervision, interpretation, writing – review and editing, project administration.

## Ethics Statement

This study was approved by the medical ethics committee in Utrecht, the Netherlands.

## Consent

Informed consent was obtained from all individual participants included in the study.

## Conflicts of Interest

The authors declare no conflicts of interest.

## Data Availability

The data that support the findings of this study are available on request from the corresponding author. The data are not publicly available due to privacy or ethical restrictions.
